# Model-based roentgen stereophotogrammetric analysis using elementary geometrical shape models: 10 years results of an uncemented acetabular cup component

**DOI:** 10.1186/s12891-018-2259-4

**Published:** 2018-09-18

**Authors:** Anne Jacobsen, Frank Seehaus, Yutong Hong, Han Cao, Alexander Schuh, Raimund Forst, Stefan Sesselmann

**Affiliations:** 1 Department of Orthopaedic Surgery, Friedrich-Alexander-Universität Erlangen-Nürnberg (FAU), Rathsberger Str. 57, 91054 Erlangen, Germany; 2 Muskuloskelettales Zentrum, Klinikum Neumarkt, Nürnberger Str. 12, 92318 Neumarkt i. d. OPf, Germany; 3 Institute for Medical Technology, Ostbayerische Technische Hochschule Amberg-Weiden, Hetzenrichter Weg 15, 92637 Weiden, Germany

**Keywords:** Total hip replacement, Acetabular cup, Implant migration, Roentgen stereophotogrammetric analysis, RSA, Elementary geometrical shapes

## Abstract

**Background:**

Non-cemented acetabular cup components demonstrated different clinical performance depending on their surface texture or bearing couple. However, clinical osseointegration needs to be proved for each total joint arthroplasty (TJA) design. Aim of this study was to detect the in vivo migration pattern of a non-cemented cup design, using model-based roentgen stereophotogrammetric analysis with elementary geometrical shape models (EGS-RSA) to calculate early cup migration.

**Methods:**

Interchangeable applicability of the model-based EGS-RSA method next to gold standard marker-based RSA method was assessed by clinical radiographs. Afterwards, in vivo acetabular cup migration for 39 patients in a maximum follow up of 120 months (10 years) was calculated using model-based EGS-RSA.

**Results:**

For the axes with the best predictive capability for acetabular cup loosening, mean (±SD) values were calculated for migration and rotation of the cup. The cup migrated 0.16 (±0.22) mm along the cranio-caudal axis after 24 months and 0.36 (±0.72) mm after 120 months, respectively. It rotated − 0.61 (±0.57) deg. about the medio-lateral axis after 24 months and − 0.53 (±0.67) deg. after 120 months, respectively.

**Conclusions:**

Interchangeable applicability of model-based EGS-RSA next to gold standard marker-based RSA method could be shown. Model-based EGS-RSA enables an in vivo migration measurement without the necessity of TJA specific surface models. Migration of the investigated acetabular cup component indicates significant migration values along all the three axes. However, migration values after the second postoperative year were within the thresholds reported in literature, indicating no risk for later aseptic component loosening of this TJA design.

## Background

The medical intervention for hip osteoarthritis finally ends with a surgical treatment of the affected joint by total hip arthroplasty (THA) [1]. Forecasts for primary and revision THA procedures predict an increasing case number [2]. However, THA presents an established orthopaedic intervention with a reported survivorship greater than 95% at 10-year and 80% at 25-year follow-up [3]. National registries [4] and clinical studies [5, 6] indicate that THA survivorship is affected by several factors like total joint arthroplasty (TJA) design, bearing couple or surface texture of non-cemented designs. In summary, THA failure most often is caused by aseptic loosening [3, 4, 7] and periprosthetic joint infection [8, 9], both associated with an important economic impact [10].

According THA, the interpretation of survivorship rates must be performed separately for femoral stem and acetabular cup components. For instance Hallan et al. [11] reported a good survivorship at 15-years follow-up for non-cemented femoral stems, whereas non-cemented metal-backed acetabular cup components revealed poor clinical outcome in comparison to cemented all-polyethylene components. Non-cemented metal backed cups showed different clinical outcomes, i.e. grit-blasted and hydroxyapatite-coated acetabular cups indicate a three times higher risk of revision than hydroxyapatite-coated, threaded acetabular cups in cause of aseptic loosening [11]. Palomäki et al. [12] reported data for uncemented cups of the second generation, collected within the Finnish Arthroplasty Register, showing survival rates similar to cemented treatment. However, these results based on mid-term data, longtime data for these cups were still necessary.

The clinical performance of non-cemented coated TJA components in general depends on several factors predicting the bony osseointegration: (*i*) properties of the implant surface likewise surface texture, coating layer thickness and applied pore size, (*ii*) host bone quality, (*iii*) surgical site preparation, (*iv*) preoperative loading conditions and (*v*) preventing initial and chronic infections [13, 14].

If the bony osseointegration of a TJA design fails, loosening of the implant will occur. Gold standard method to assess the in vivo fixation of TJA components presents Roentgen Stereophotogrammetric Analysis (RSA) [15, 16]. It is important to monitor TJA fixation within preclinical trials using gold standard methods, since worst case scenarios of clinical TJA failure – likewise the Boneloc cement disaster within the early 1990s – could be avoided [17–20] and the migration pattern of TJA is affected by the implant design and its fixation philosophy [21, 22]. Using RSA, early implant migration within the first two postoperative years could be detected, which has been shown to correlate well with later aseptic loosening of femoral stems [23] and acetabular cups [24, 25].

Several variations of RSA exist, i.e. using markers attached to the implant (marker-based RSA) or a surface model of the investigated TJA components (model-based RSA) to calculate early implant migration [20, 26]. The interchangeable applicability of the both methods has been demonstrated [27–29]. Model-based RSA using elementary geometrical shape (EGS-RSA) is an approach which uses geometrical bodies likewise spheres or cylinders to calculate migration by rigid body kinematics instead of implant specific surface models [30–33].

Nieuwenhuijse et al. [24] identified thresholds for micromotion of a cemented acetabular cup to distinguish between safe or at risk implants. This research group found cranial migration > 1.76 mm or sagittal rotational migration > 2.53 deg. within the first two postoperative years to be risk factors for subsequent aseptic component failure of acetabular cups. Although a cemented cup was investigated, the authors state that the results of their study should be independent of the fixation method [24]. In accordance with this statement, Pijls et al. [25] reported in a systematic review a similar threshold for cemented as well as uncemented cups. A mean cranial migration of 0.2 mm or less was classified as acceptable, with revision rates of < 5% after 10 years.

Aim of this study is to determine the in vivo migration behavior of a non-cemented titanium cup (*Phoenix cup*; Peter Brehm GmbH, Weisendorf, Germany) for 10 years, using model-based EGS-RSA. The authors hypothesize that no translational migration > 1.7 mm in cranial direction or rotational migration > 2.5 deg. around anterior-posterior axis is detectable within a two-year follow-up. Thus, the authors expect good ten-year performance of the cup with a revision rate of less than 5% caused by aseptic implant failure.

## Methods

**Fig. 1:**
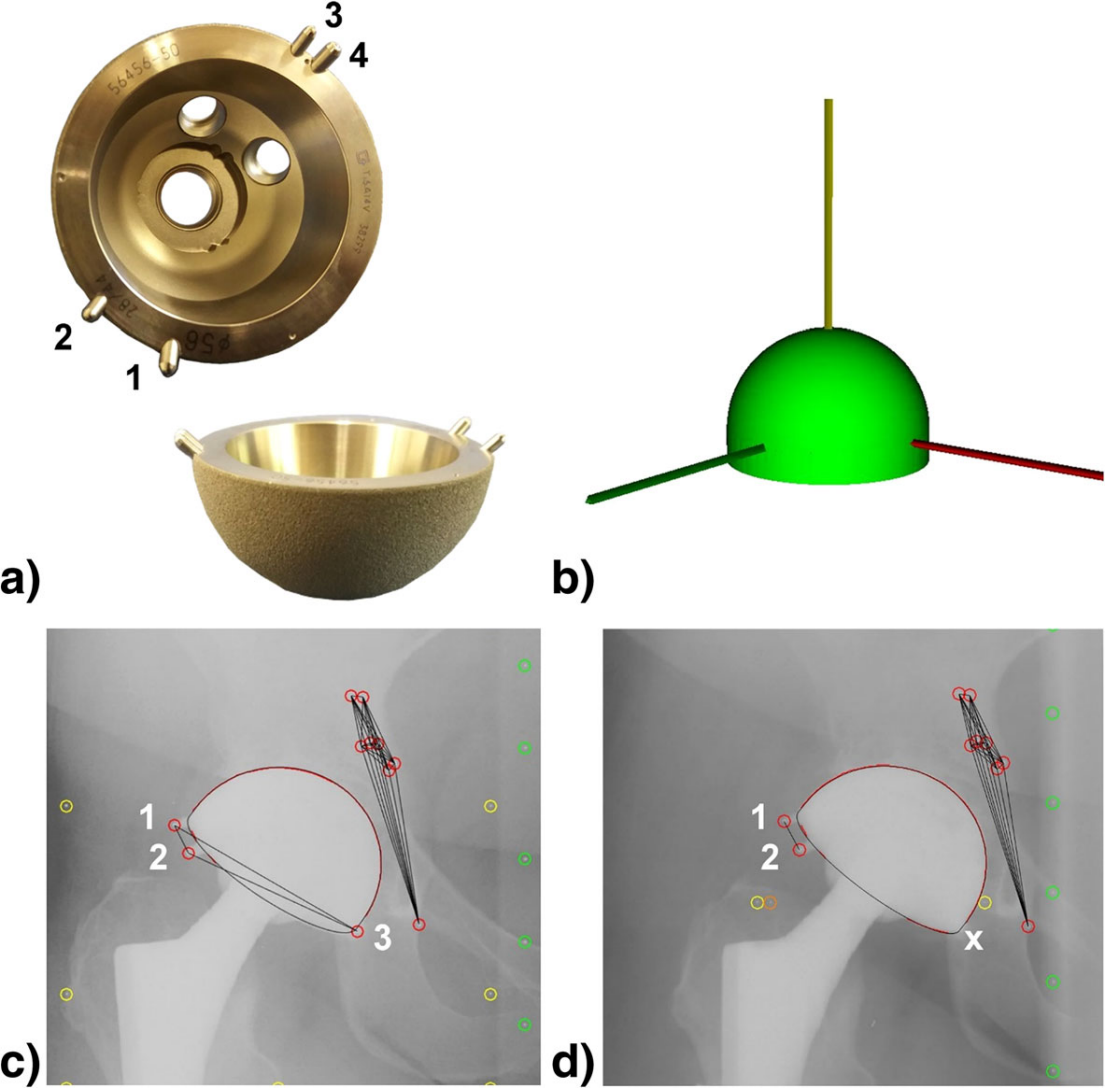
**a** Non-cemented titanium (TiAl_6_V_4_) acetabular cup component, composed of a cylinder attached to the rim of a spherical segment, covered by TiRC coating (Titan Rough Coating) with a surface layer thickness of approximately 300 μm, a surface roughness of R_z_ = 100–150 μm and four additional attached and pairwise-arranged tantalum markers for marker-based RSA measurements. This special RSA cup was in accordance with legal rules. **b** For model-based EGS-RSA approach, a hemisphere model with a curvature radius and implant height according to the technical drawings of the investigated cup component was generated. The local EGS model coordinate system was set to the centre of gravity. All axes were aligned perpendicular to each other. **c** Left image of an RSA reference radiographic image pair with three of four visible implant markers. Implant marker no. 4 occluded by the implant itself within the RSA image. **d** Occluded implant marker problem for marker-based RSA approach. Left image out of the same patient follow up series, with occluded implant markers no. 3 (x) and 4. As at least three implant markers were necessary for marker-based RSA approach, for this RSA image pair a migration calculation was not possible

Retrospectively, out of a former classical marker-based RSA study of 50 patients (Fig. 1a), RSA radiographs were analysed using model-based RSA approach with a hemisphere elementary geometrical shape surface model (Fig. 1b). Marker-based migration calculation was not possible due to implant-marker occlusion problems (in 43 of 50 cases) within the RSA radiographs (Fig. 1c, d). But using a model-based RSA approach with EGS models, migration calculation was possible in 39 of 50 cases.

### Surgical treatment

All included patients showed clinical signs of hip osteoarthritis and were treated by primary THA with a non-cemented titanium acetabular cup component (*Phoenix cup*, Peter Brehm GmbH, Weisendorf, Germany) (Fig. 1a). The *Phoenix cup* is a hemispherical press-fit cup with a titanium rough coating and a surface roughness of Rz 100–150 μm. A single well skilled surgeon performed all the surgeries, using a direct lateral approach. For each patient six to eight CE-certified tantalum markers (diameter: 1.0 mm) were inserted during surgery to the implant surrounding acetabular bone.

### RSA measurement setup

A uniplanar RSA setup consisting of two x-ray tubes (*Multix RD 82477–01 Vertix ACS* and *Mobilett Plus*; Siemens, Berlin, Germany) was used. Both x-ray tubes were positioned at an angle of 40 deg. in relation to each other and 1.40 m above the calibration cage (*Umea Cage 43 calibration cage*, RSA BioMedical Innovations AB, Umea, Sweden). The THA treated hip joints of the supine patients were arranged within the intersection of the two x-ray pathways. Accuracy of this RSA setup was determined using a custom made phantom model (Table 1), as double examinations were declined by the ethical committee to minimize x-ray exposure of the patients.

**Table 1:** Accuracy of the investigated acetabular cup component within a phantom-model and a performed relative motion protocol

	RSA Translation [mm]			EGS Translation [mm]			set-point [mm]
	*x*	*y*	*z*	*x*	*y*	*z*	
mean	0.50	0.48	0.51	0.49	0.48	0.44	0.5
SD	0.03	0.05	0.08	0.03	0.05	0.18	
bias	0.00	−0.02	0.01	−0.01	− 0.02	− 0.06	
95% CI (↓)	−0.05	− 0.08	− 0.12	−0.04	− 0.08	−0.27	
95% CI (↑)	0.08	0.13	0.20	0.06	0.13	0.43	
mean	1.49	1.47	1.52	1.49	1.47	1.49	1.5
SD	0.02	0.06	0.05	0.04	0.06	0.25	
bias	−0.01	−0.03	0.02	−0.01	−0.03	−0.01	
95% CI (↓)	−0.01	0.00	−0.02	−0.02	− 0.03	−0.12	
95% CI (↑)	0.08	0.22	0.17	0.15	0.20	0.86	

After taking RSA image pairs of the reference setup, using a spring-loaded micrometer, stepwise increments of 0.5 up to 1.5 mm (set-point value for single translation along each axis) of the cup along the x-, y- and z-axis was performed. At each step a RSA image pair was taken. This protocol was repeated *n* = 8 times. RSA image pairs were analysed by marker-based RSA and model-based EGS-RSA

*SD* standard deviation*, bias* difference between mean value and set point value, *CI* confidence interval with upper (↑) and lower (↓) limit

### Measurement protocol

Reference RSA image pairs were taken within the first two postoperative days before load bearing of the treated hip joint. Follow-up examinations were performed at six, 12 weeks, six, 12, 24, 60 and 120 months postoperatively.

The RSA image pairs of all patients (*n* = 50) were analysed using model-based RSA software package (*MBRSA 3.4*, MEDIS specials, Leiden, Netherlands) and custom made EGS models of the investigated acetabular cup component (Fig. 1b). Standard thresholds were applied to verify the quality of the calibration procedure and the rigid body error of the detected bone markers of the acetabular bone [34–36]. If all quality criteria were fulfilled in the image pairs, these datasets were included for statistical analysis, indicating that no artefacts or analysis errors were present. Only patients with at least one follow-up at 2 years, 5 years or 10 years were included.

RSA image analyses were performed in accordance with the recommendations of the RSA ISO standard 16,087:2013(E) [36] and the guidelines proposed by Valstar et al. [34]. All patients gave their written informed consent to study participation and to data publication. The Ethics Committee of Friedrich-Alexander-Universität Erlangen-Nürnberg approved the study (registration no. 1.077).

### Statistics

Calculated migrations of clinical data were presented as mean (±SD). An analysis of variance including Bonferroni correction was used to prove significant changes of migration values between the follow-ups. The level of significance was set to *p* < 0.05. Statistical analyses were performed using SPSS 24.0 (*SPSS Inc.*, Chicago, Illinois, USA).

## Results

### Patient cohort

**Fig. 2:**
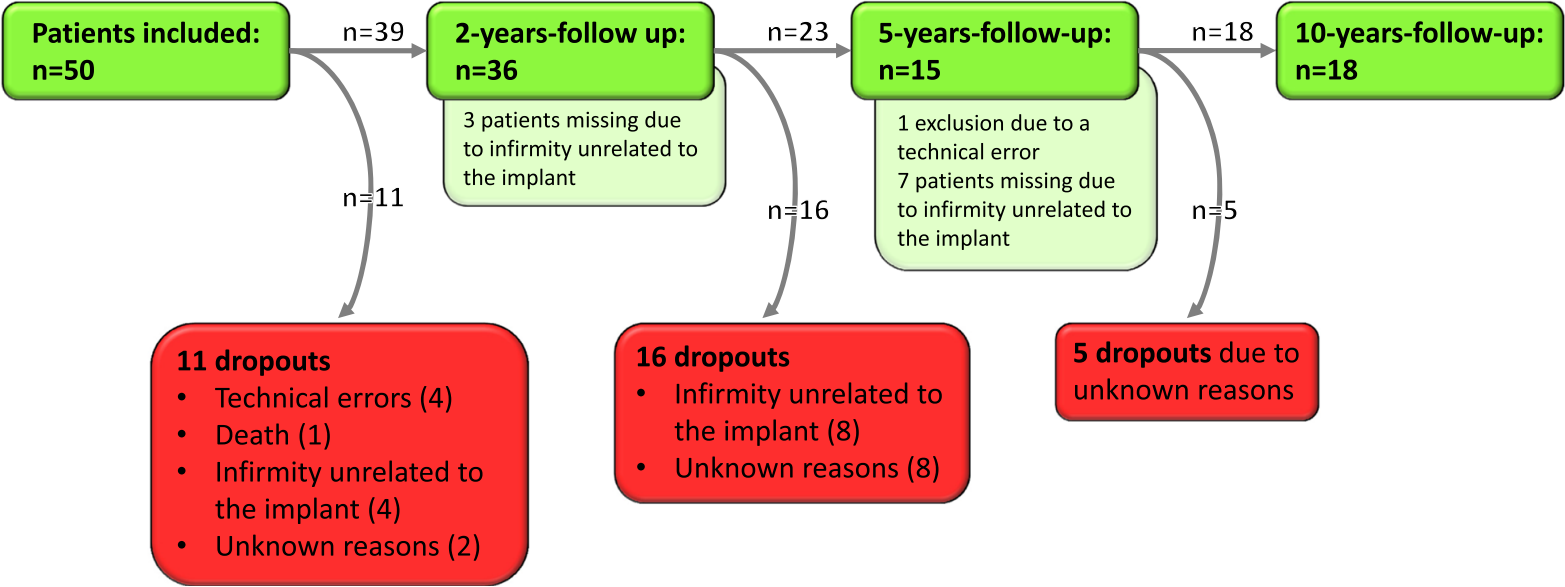
Flowchart of dropouts and missing patients. Green boxes: attending patients on follow-up. Pale green boxes: patients missing the correspondent follow-up. Red boxes: final dropouts

Within this retrospective analysis, 11 patients of the entire patient cohort (*n* = 50) had to be excluded due to technical errors (e.g. high rigid body error of the bone markers at the postoperative) (*n* = 4), death (*n* = 1), study exclusion for reasons unrelated to the implant (*n* = 4) or for unknown reasons (*n* = 2). In summary radiographs for 39 patients, which completely fulfilled analysis criteria for model-based EGS-RSA measurement were included. After the 2-years follow-up, 16 patients dropped out of the study for infirmity not related to the implant (*n* = 8) or for unknown reasons (*n* = 8), as it was not possible to contact the latter patients. After the 5-years follow-up, again five more patients got lost to the study for unknown reasons (Fig. 2).

The entire patient cohort (*n* = 39) consisted of 24 women and 15 men (18 left and 21 right hip replacements). The mean age of the patients at time of surgery was 67.2 (±9.5) years, ranging from 35.8 to 79.6 years.

### Experimental accuracy of marker- and model-based EGS-RSA for the investigated cup component

Maximum bias (±SD) for marker-based RSA was − 0.03 (±0.06) mm (95% CI between < 0.01 and 0.22 mm) in cranio-caudal direction (y-axis), for model-based EGS-RSA, it was − 0.06 (±0.18) mm (95% CI between − 0.27 and 0.43 mm) along anterior-posterior direction (z-axis), for the both investigated relative motion set points of 0.5 mm and 1.5 mm (Table 1).

### Clinical cup migration data

Highest mean (±SD) migrations were observable along medio-lateral direction (x-axis) with 0.29 (±0.52) mm at 60 months and along cranio-caudal direction (y-axis) with 0.36 (±0.27) mm at 120 months (Table 2). Rotational migration about the y-axis was not calculated according to axisymmetric implant geometry. For the remaining axes the highest rotation occurred around medio-lateral direction of − 0.61 (±0.57) deg. at 24 months follow-up (Table 2).

**Table 2:** Non-cemented titanium acetabular cup component migration – mean and standard deviation values at 6, 12 weeks, 6, 12, 24, 60 and 120 months follow-up using model-based EGS are presented. According to axis-symmetric implant design of the cup component, Ry migration values are not given

Follow-up	Translation [mm]			Rotation [deg]			Cases [n]
	*x*	*y*	*z*	*Rx*	*Ry*	*Rz*	
Reference							
mean	0.00	0.00	0.00	0.00	–	0.00	39
SD	0.00	0.00	0.00	0.00	–	0.00	
6 weeks							
mean	0.20	0.11	−0.04	−0.17	–	−0.10	32
SD	0.41	0.22	0.33	0.96	–	0.72	
12 weeks							
mean	0.16	0.14	−0.06	−0.12	–	−0.08	33
SD	0.44	0.23	0.39	0.86	–	0.80	
6 months							
mean	0.22	^*^ **0.16**	0.03	−0.27	–	0.05	37
SD	0.45	0.25	0.42	0.95	–	0.76	
12 months							
mean	0.23	^*^ **0.16**	0.07	−0.30	–	−0.19	39
SD	0.44	0.22	0.38	0.91	–	0.67	
24 months							
mean	0.28	^*^ **0.17**	0.02	^*^ **-0.61**	–	0.00	36
SD	0.46	0.24	0.50	0.57	–	1.03	
60 months							
mean	0.29	0.19	−0.02	−0.21	–	− 0.07	15
SD	0.52	0.27	0.43	1.24	–	0.47	
120 months							
mean	0.19	^*^ **0.36**	−0.04	− 0.53	–	0.22	18
SD	0.69	0.27	0.35	0.67	–	1.21	

*SD* standard deviation

^*^mean value significant different (*p* < 0.05) to reference results

For the most predictable axis of acetabular cup loosening, migration along cranio-caudal axis increased continuously from 0.11 (±0.22) mm at 6 weeks up to 0.36 (±0.27) mm at 120 months.

**Fig. 3:**
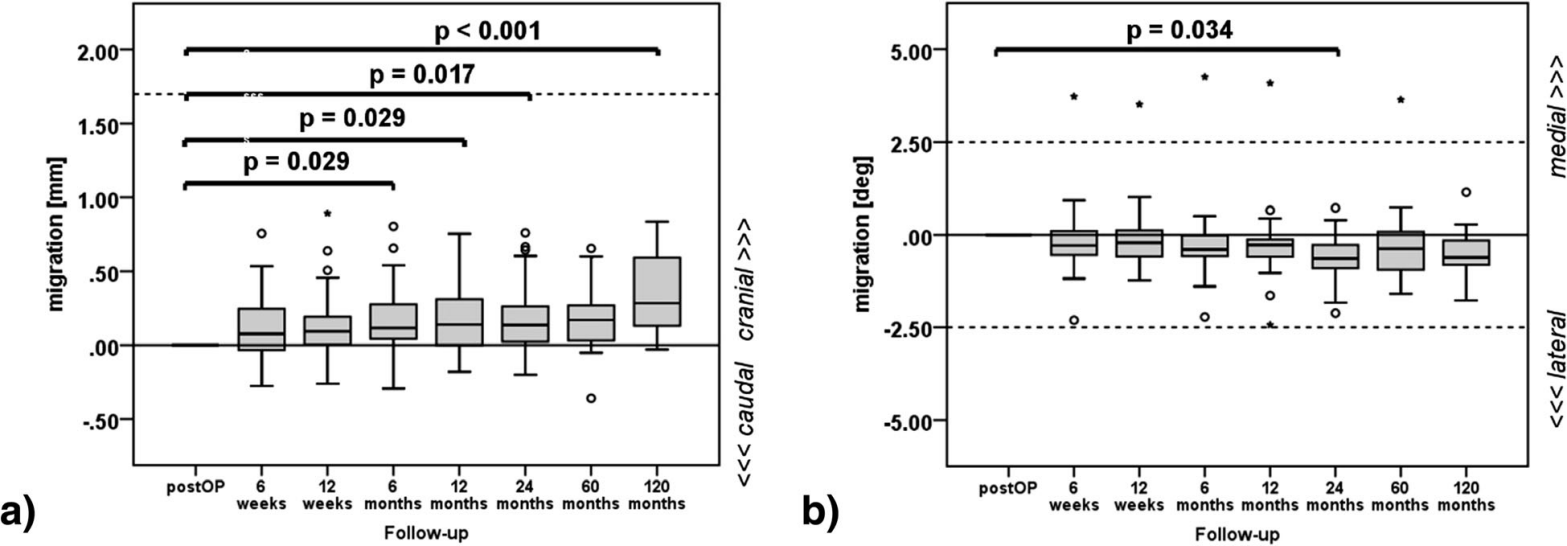
Calculated acetabular cup migration (**a**) along cranio-caudal direction [mm] and (**b**) about medio-lateral direction [deg] at six, 12 weeks, six, 12, 24, 60 and 120 months follow-up. The boxes represent 50% of included data (bound the 25th to 75th percentile) with the median (horizontal line), smallest and largest value (whisker bars) as well as outliers (circles) and extreme values (stars). Dotted horizontal lines indicate threshold for cranial (> 1.76 mm) acetabular cup migration according to Nieuwenhuijse et al. [24]

In cranio-caudal direction significant migration values according to the reference follow-up examination were observable for the 6 months follow-up (*p* = 0.029), 12 months follow-up (*p* = 0.029), 24 months follow-up (*p* = 0.017) and 120 months follow-up (*p* < 0.001). However, for the 60 months follow-up (*p* = 0.114) no significant difference to the reference follow-up could be observed (Fig. 3a, Table 2). For rotation about medio-lateral axis a significant different mean (*p* = 0.034) compared to the reference follow up was observable at 24 months (Fig. 3b, Table 2). For remaining migrations and rotations, significance criteria were not fulfilled.

## Discussion

Accuracy determined by using a phantom investigation showed better values for the marker-based RSA than for the model-based EGS-RSA method. However, both methods provided results within the range of RSA accuracy reported by Kärrholm [37], which is between 0.05 and 0.5 mm for translational migration. Thus, we consider that the loss of accuracy from using model-based EGS-RSA is acceptable for a clinical application of the methodology.

The clinical outcome of the investigated cup component indicates statistically significant migration according to the reference examination along the cranio-caudal axis at the 6 months (0.16 mm; *p* = 0.029), 12 months (0.16 mm; *p* = 0.029) and 24 months follow-up (0.17 mm; *p* = 0.017). At 120 months follow up, migration of the cup component increased significantly up to 0.36 (±0.27) mm (*p* < 0.001). It is important to note that in the first 24 months the observable maximum mean migration in cranial (0.11 mm) direction occurred within the first 6 weeks after surgery (Table 2). Afterwards a steady state mode of the detected migration was observable for the upcoming follow-up intervals within the first 5 years. Between five and 10 years the cup continued to migrate in cranial direction again by another 0.17 mm.

The only observable significant different rotation occurred around the medio-lateral axis at the 24 months follow-up (− 0.61 deg.; *p* = 0.034).

**Fig. 4:**
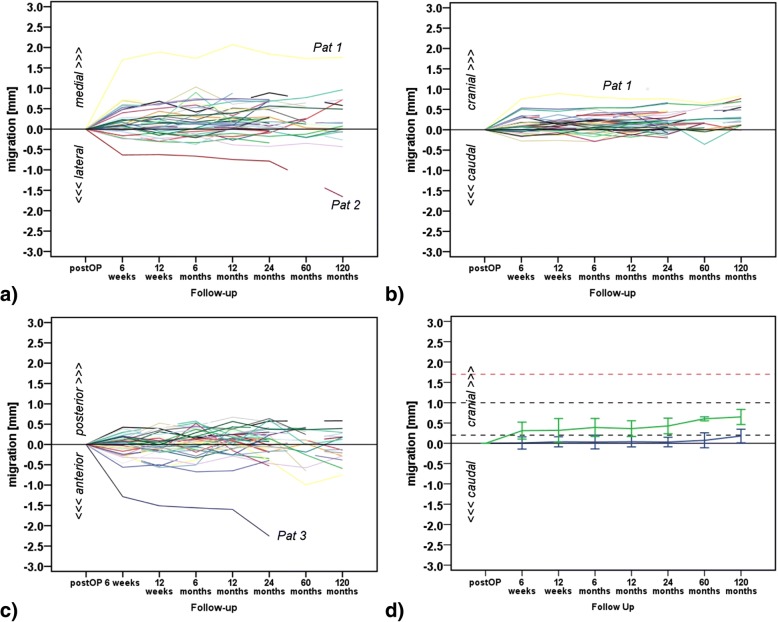
Calculated acetabular cup migration [mm] at six, 12 weeks, six, 12, 24, 60 and 120 months follow-up along (**a**) medio-lateral, **b** cranio-caudal and (**c**) anterior-posterior direction. **d** Mean ± SD value for each patient, classified at 24 months follow up according to Pijls et al. [25] as an acceptable cup (blue graph; migration < 0.2 mm represented by black dotted line) and cup at risk (green graph; migration between 0.2 mm and 1.0 mm represented by black dotted line). Red dotted line indicates threshold (> 1.76 mm) for cranial acetabular cups migration at risk according to Nieuwenhuijse et al. [24]

Two patients showed especially noticeable migration greater than 1.0 mm in the medial direction (Pat 1; 1.7 mm) and of approximately 1.0 mm in lateral direction (Pat 2) within the first 6 weeks, after which the cup appeared to stabilise up to the 24 months follow up (Fig. 4a). However, Pat 2 cup starts to migrate again in lateral direction after 24 months follow up. Conventional anterior-posterior radiographs indicate no clinical signs of loosening (e.g. radiolucent lines).

Some patients (*n* = 13 at 24 months; *n* = 11 at 120 months, including Pat 1) showed migration between 0.2 mm and 1.0 mm in cranial direction (Fig. 4b), however no cups were identified to migrate more than 1.0 mm. The maximum cranial migration value was observable after 6 weeks with 0.76 mm (Pat 1), which remained unchanged for this patient during the next follow-up investigations. Thus, this cup seems to have stabilised with no further migration in this direction.

In anterior-posterior direction one patient (Pat 3) had a migration value of − 1.28 mm at 6 weeks follow-up, which increased to > − 2.0 mm at the 24 months follow up (Fig. 4c).

Cranial migration is seen as a relevant predictive value for late aseptic loosening of acetabular cup components [24, 25, 38, 39]. In a systematic review, Pijls et al. [25] reported an average increase of 10% in the 10-year revision rate for every millimetre of cranial migration of an acetabular cup component. The authors suggested that inferior components could be identified by RSA within the first two postoperative years, thereby avoiding their widespread implementation. Furthermore, they provided proximal migration thresholds for acetabular cups, whereas cups with a mean cranial migration of 0.2 mm or less were classified as acceptable, with revision rates of < 5% after 10 years. In contrast, cups with a mean cranial migration of 1.0 mm or more were classified as unacceptable, with a > 5% risk for 10-year revision.

For the investigated cup component, a mean proximal migration of 0.17 mm occurred after two postoperative years (*n* = 36 patients at 24 months), which is less than the proposed threshold of 0.2 mm suggested by Pijls et al. [25]. In summary, 13 patients at 24 months were classified at risk (migration between 0.2 mm and 1.0 mm), no patient could be classified as unacceptable (migration > 1.0 mm). These results indicate good performance for the investigated cup component, according to suggestion of Pijls et al. [25]. As none of the followed implants had to be revised after 10 years, this study verifies the suggestions of Pijls et al. [25].

One limitation of this study was the absence of rotational measurements about the cranio-caudal axis. Due to technical restrictions of EGS-RSA, analysis of this rotational migration could not be conducted as the cup has an axis-symmetrical design. This is a well-known problem in model-based RSA [40]. Nonetheless, migration in cranial direction and rotation about the anterior-posterior axis could be calculated, which are the essential movements to predict later cup loosening [24, 25]. Current studies evaluate computed tomography (CT) as an alternative measurement tool to RSA to analyze implant migration. They describe comparable accuracy and precision values for CT-based methods, especially in experimental settings [41–44]. Moreover, CT-based migration measurement could handle some disadvantages of RSA: CT is available in almost every hospital, picture acquisition does not require a special setup or briefed personnel, migration in all directions can be calculated, and there is no problem of marker occlusion by the implant [45]. On the other hand, conventional CT examinations expose patients to higher radiation doses than stereoradiographs, but there is some evidence that a reduction of CT radiation dose does not impair the accuracy of migration analysis [42, 43]. However, there is still a lack of clinical trials and long-term examinations evaluating CT-based migration measurement. Besides, there are still some problems to deal with, as for example artifacts of metallic implants, the influence of soft tissue or the immobilization of patients during CT scanning [44]. In summary, CT-based methods need further validation before they can be considered as an alternative to gold standard RSA.

Another limitation of this study is the number of drop-outs. However, the drop-out rate is comparable to those of other RSA studies [23, 24, 46–48]. During the crucial period of the first 2 years, that are relevant for RSA-prognosis, eleven patients had to be excluded. The remaining number of 39 patients also complies with the RSA guidelines proposed by Valstar et al., who recommend a number of 15–25 patients for the special case of RSA studies [34].

Unfortunately, it is not possible to define prognostic migration thresholds out of the results, as there was no implant failure observable in 10 years of follow-up. However, the results confirm the above-mentioned thresholds of previous studies.

The migration pattern of the investigated acetabular cup component showed migration along all the three axes, particularly in medio-lateral and cranio-caudal direction. Migration along the latter mentioned direction is considered to be the most important prognostic factor for a later aseptic loosening but was still within acceptable limits according to Pijls et al. [25]. It would be interesting to follow the patients for a longer period in order to evaluate how the migration pattern of all axes will influence the long-term stability of the investigated non-cemented acetabular cup.

## Conclusions

Model-based EGS-RSA enables an in vivo migration measurement of TJA components without the necessity of TJA specific surface models. The investigated acetabular cup component demonstrates significant differences for calculated migration values along the most predictable axis of acetabular cup loosening, migration along cranio-caudal axis with 0.17 (±0.24) mm at 24 months. However, the measured in vivo cup migration at 2 years of follow-up were within the acceptable thresholds reported in literature [24, 25]. For the cranio-caudal axis, migration continuously increased up to 0.36 (±0.27) mm at 120 months follow-up but is still within above mentioned thresholds. However, performed migration measurement by model-based EGS-RSA within this study highlights the importance and benefit of RSA for the evaluation of implants. There may be other possible applications in the future, such as the early detection of septic loosening of joint implants [49].

## Abbreviations

EGS - RSA: Roentgen stereophotogrammetric analysis using elementary geometrical shape models
CT: Computed tomography
EGS: Elementary geometrical shapes
RSA: Roentgen stereophotogrammetric analysis
SD: Standard deviation
THA: Total hip arthroplasty
TJA: Total joint arthroplasty
